# Evaluating temozolomide for pediatric adamantinomatous craniopharyngiomas using microspheroid-based drug screening

**DOI:** 10.1007/s00381-026-07161-8

**Published:** 2026-02-27

**Authors:** Sérgio Cavalheiro, Lorena Favaro Pavon, Caroline Brunetto de Farias, Nasjla Saba da Silva, Flávia Borelli Nascimento, Tatiana Tais Sibov, Jessica Benigno Rodrigues, Patrícia Alesssandra Dastoli, Fernando Seiji Suzuki, Rodrigo Akira Watanabe, Isaque Hyung Tong Kim, Martina Lichtenfels, Camila Alves da Silva, Andrea Cappellano, Marcos Devanir Silva da Costa

**Affiliations:** 1 Translacional Neurosurgery Laboratory, Department of Neurology and Neurosurgery, Universidade Federal de Sao Paulo (UNIFESP), São Paulo, SP Brazil; 2 Pediatric Oncology Institute, Grupo de Apoio Ao Adolescente E À Criança Com Câncer (GRAACC), Universidade Federal de Sao Paulo (UNIFESP), São Paulo, SP Brazil; 3 National Science and Technology Institute for Children’s Cancer Biology and Pediatric Oncology – INCT BioOncoPed, Porto Alegre, Brazil; 4 Department of Translational Research, Ziel Biosciences, Porto Alegre, Rio Grande Do Sul, Brazil; 5 Postgraduate Program in Biological Sciences: Pharmacology and Therapeutics, Universidade Federal Do Rio Grande Do Sul, Porto Alegre, RS Brazil; 6 Postgraduate Program in Maternal and Child Health of the Institute of Pediatrics, Universidade Federal Do Rio de Janeiro, Rio de Janeiro, RJ Brazil

**Keywords:** Pediatric neuro-oncology, Craniopharyngiomas, MGMT expression, Chemoresistance, Hypothalamus

## Abstract

**Purpose:**

Pediatric adamantinomatous craniopharyngioma (ACP) is the most common tumor of the diencephalic–pituitary axis in children. Although histologically benign, pediatric ACP is frequently associated with substantial endocrine dysfunction and neurological complications. The gold-standard treatment is complete surgical resection; however, because of the tumor’s location, total removal is often not feasible. Consequently, several authors recommend radiotherapy as an adjuvant option. Nevertheless, partial resections are frequently followed by recurrence, and repeated surgical interventions increase morbidity and impair quality of life. Thus, adjuvant therapeutic strategies capable of controlling this tumor should be encouraged.

**Methods:**

We analyzed seven fresh tumor specimens ACP from patients < 18 years of age using a chemoresistance platform (*Bioverso Test*,* Ziel Biosciences*, São Paulo, Brazil). These cases demonstrated widespread resistance to most chemotherapeutic agents tested. Temozolomide (500 µM) was the only drug that showed consistent and significant sensitivity.

**Results:**

Based on these findings, we initiated treatment in a 14-year-old patient with recurrent ACP who had previously undergone multiple surgical procedures and radiotherapy. The tumor involved the left cavernous sinus and extended into the sphenoid sinus. Clinically, the patient was amaurotic and presented with panhypopituitarism. The patient received temozolomide (200 mg/m^2^/day for 5 consecutive days in 28-day cycles). After completing 12 cycles of chemotherapy, there was a notable regression of the lesion, with approximately 50% reduction in total tumor volume.

**Conclusion:**

These findings suggest that temozolomide may represent a promising therapeutic option for controlling ACP.

## Introduction

Craniopharyngioma is a histologically benign epithelial neoplasm with limited genetic alterations. It exhibits heterogeneous radiological and pathological features, including solid, cystic, or mixed solid-cystic components, with or without calcifications, and is associated with high recurrence rates [1, 34, 43]. This tumor accounts for approximately 1–3% of all intracranial tumors and represents the most common non-neuroepithelial intracranial neoplasm in childhood [23, 49]. Two distinct histological subtypes have been described, each with unique signaling pathways: (i) adamantinomatous craniopharyngioma (ACP), which is prevalent in children (92–96% of pediatric cases) and driven by activation of the WNT/β-catenin pathway, and (ii) papillary craniopharyngioma, which is rare in children and more common in adults [23], associated with alterations in the BRAF and MEK inhibition pathways [26, 27]. Although complete surgical resection is considered curative, recurrence rates remain substantial, with up to 40% of cases relapsing despite total resection [2, 10, 19]. Consequently, several alternative therapeutic strategies have been proposed to improve disease control, including immunotherapy, radiotherapy, gamma knife radiosurgery, cyst aspiration, intracystic chemotherapy, and targeted therapies [6, 32, 33, 46].

Recent studies have identified novel ACP molecular subtypes [3], revealing critical signaling pathways involved in ACP development and progression/β-catenin, TGF-β, and MAPK/ERK. The tumor microenvironment is modulated by factors such as cellular senescence, immunomodulatory cytokines, and matrix metalloproteinases (MMPs). These discoveries have enabled the classification of ACP into three molecular subtypes: WNT type, immune group A (ImA), and immune group B (ImB) [4, 17, 20, 50], providing a framework for potential personalized therapeutic strategies.

Despite this progress, there remains a pressing need for effective, low-cost chemotherapeutic agents capable of controlling tumor growth, reducing recurrence, and mitigating associated complications. This study was conducted to evaluate the potential utility of temozolomide as a chemotherapeutic option for ACP management.

## Material and methods

Between September 2021 and January 2024, tumor specimens from 50 patients < 18 years of age with central nervous system (CNS) neoplasms who underwent surgical resection at the Institute of Pediatric Oncology – Grupo de Apoio ao Adolescente e à Criança com Câncer (GRAACC) were subjected to chemoresistance profiling using the Bioverso Test_Ziel platform(*Biosciences*, São Paulo, Brazil). This 96-well plate system is preloaded with chemotherapeutic agents commonly used in neuro-oncology and validated by the Translational Neurosurgery Laboratory of the Paulista School of Medicine–Federal University of São Paulo. Ethical approval was obtained from the GRAACC Ethics Committee (Approval No. 0384/2022) and the Research Ethics Committee of the Universidade Federal de São Paulo–UNIFESP (CAAE: 58018222.2.0000.5505). All procedures followed the Declaration of Helsinki.

The platform was validated using CNS tumor cell lines (U-251, M059J, A172, U87MG) from the *American Type Culture Collection*, representing aggressive and treatment-refractory phenotypes. Each line was exposed to multiple drug concentrations to generate resistance curves. Agents included bleomycin, carboplatin, cyclophosphamide, cisplatin, etoposide, irinotecan, lomustine, metformin, vinblastine, vincristine, bevacizumab, topotecan, dactinomycin, and temozolomide. All compounds were stabilized at supratherapeutic doses derived from IC_50_ references, using tenfold increases to ensure negative predictive validity rather than mimic in vivo exposure. This approach improves drug stability and assay reliability.

ACP tumor specimens > 1.0 cm^3^ were fragmented into < 1 mm^3^ pieces and enzymatically dissociated with trypsin, collagenase, DNase, and hyaluronidase. After density gradient separation (Histopaque), suspensions were maintained under agitation at 37 °C and 5% CO_2_ for 2 h, then filtered through 70–100 µm meshes. Microspheroids were plated at 18 × 10^3^ cells per well, in triplicate, on the Bioverso Test platform. Cultures were maintained for 72 h in DMEM-LG with 10% fetal bovine serum, 1% antibiotic–antimycotic solution, and 200 mM l-glutamine at 37 °C with 5% CO_2_.

Next, 11 µL of MTT solution (5 mg/mL) was added to each well for 2 h. After aspiration, 100 µL of dimethyl sulfoxide (DMSO) was added for 30 min. Optical density at 570 nm was measured using an ELISA plate reader. Viability was calculated relative to untreated controls and categorized as low (< 40%), medium (40–60%), or high resistance (> 60%), where low viability reflected greater drug sensitivity.

## Results

**Fig. 1:**
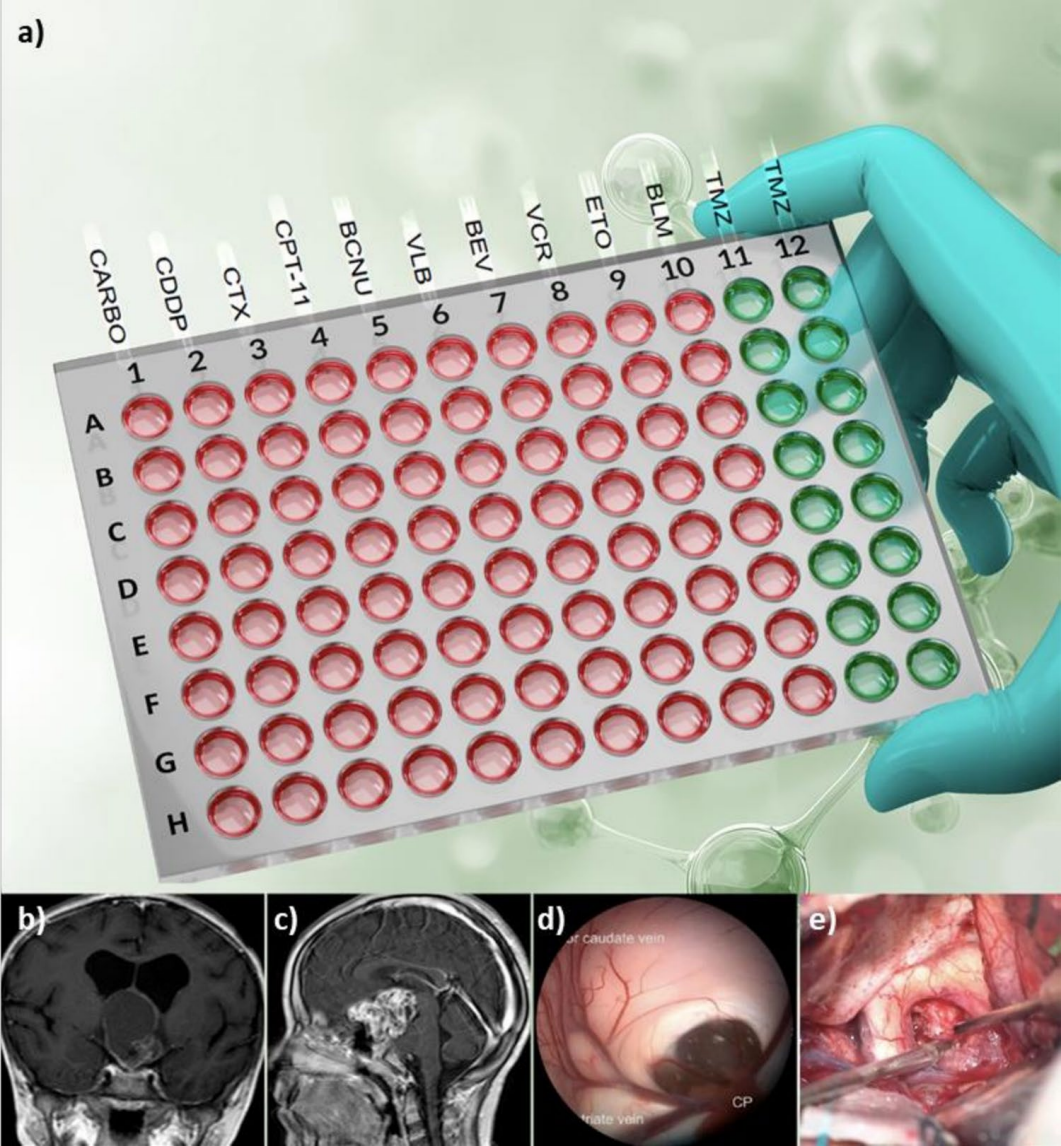
**a** Schematic representation of the 96-well chemoresistance platform (Bioverso Test) used to evaluate adamantinomatous craniopharyngioma (ACP) samples, demonstrating high sensitivity to temozolomide (TMZ, indicated in green) and resistance to other tested chemotherapeutic agents (indicated in red). **b**, **c** Magnetic resonance imaging of ACP lesions. **d** Intraoperative endoscopic view of an ACP obstructing the foramen of Monro. **e** Trans-lamina-terminalis sub-frontal access visualizing the optic chiasm and calcified ACP

Chemoresistance testing was successfully performed in all 50 CNS tumor specimens. Most results were consistent with the chemotherapeutic regimens prescribed by the oncology team. Findings in seven ACPs of the 50 CNS tumors were notably distinct. All ACP specimens showed high sensitivity to temozolomide at 250 µM and 500 µM (Fig. 1a). No papillary craniopharyngiomas were identified.

**Fig. 2:**
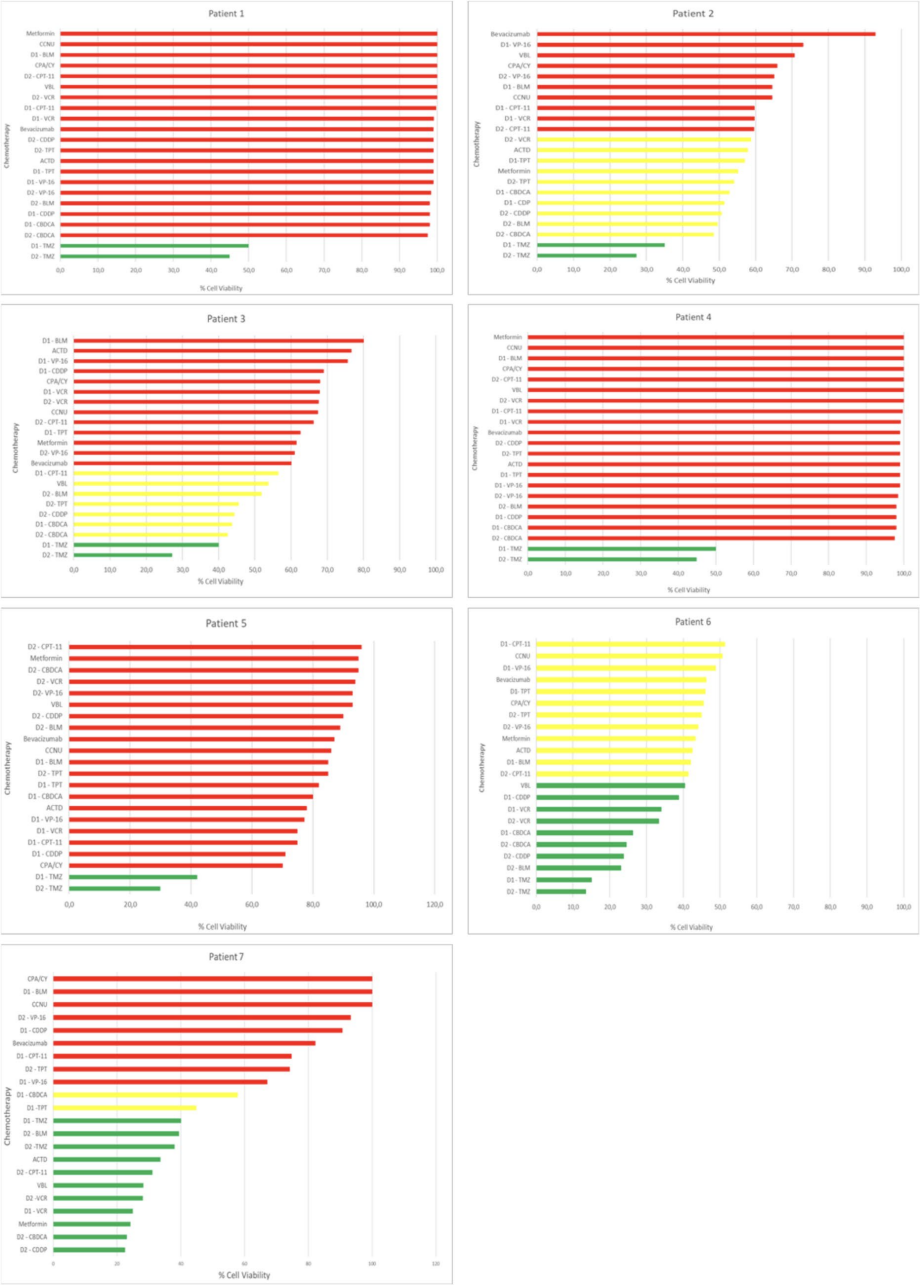
Results of chemoresistance testing in patients with ACPs (*n* = 7), showing cell-viability percentages in response to several chemotherapeutic agents: temozolomide (TMZ), topotecan (TPT), carboplatin (CBDCA), cisplatin (CDDP), bleomycin (BLM), vinblastine (VBL), irinotecan (CPT-11), etoposide (VP-16), vincristine (VCR), lomustine (CCNU), cyclophosphamide (CPA/CY), dactinomycin (ACTD), bevacizumab, and metformin. Resistance profiles are categorized as low (< 40%, green), intermediate (40–60%, yellow), and high (> 60%, red), with responses at D2 (500 µM) generally exceeding those at D1 (250 µM). ACP, adamantinomatous craniopharyngioma

Surgical resection was performed for all ACPs; four procedures were transsphenoidal and three were performed via craniotomy. The mean patient age was 9.5 years, and three patients were female. Six patients were treatment-naive, and one had undergone two surgeries and radiotherapy. All seven patients demonstrated marked sensitivity to temozolomide, with varying percentages of viable cells (Fig. 2).

In two cases (patients 6 and 7), additional agents showed sensitivity alongside temozolomide, including bleomycin, vinblastine, irinotecan, vincristine, cisplatin, dactinomycin, and carboplatin. All patients exhibited low expression of O-6-methylguanine-DNA methyltransferase (MGMT), a DNA repair enzyme associated with temozolomide resistance (Table 1).

**Table 1:** Characteristics of patients with adamantinomatous craniopharyngioma included in the study

Patient information			Clinical data			
Patients	Age	Sex	1 st surgery	2nd surgery	PR	MGMT
1	12	M	+	-	-	-
2	8	F	+	-	-	-
3	11	M	+	-	-	-
4	6	F	+	-	-	-
5	5	M	+	+	+	-
6	13	F	+	-	+	-
7	12	M	+	-	-	-

Characteristics of samples obtained from patients with ACP

*ACP* adamantinomatous craniopharyngioma, *M* male, *F* female, *PR* previous radiotherapy, *MGMT* low/negative immunoreactivity to O-6-methylguanine-DNA methyltransferase (–)

**Fig. 3:**
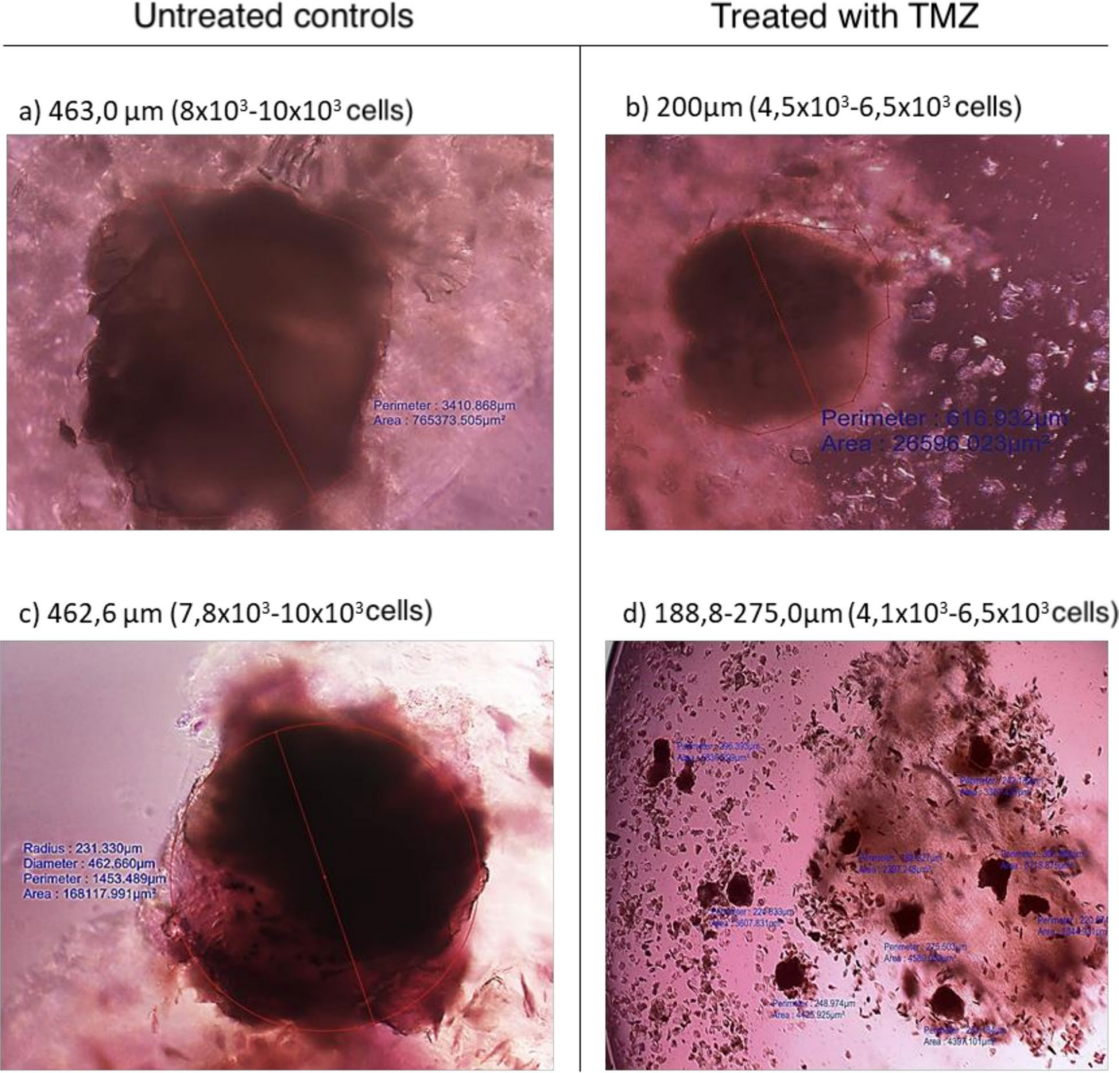
Morphological and volumetric analysis of ACP microspheroids comparing untreated controls (**a**, **c**) with microspheroids treated with temozolomide at D2 (500 µM) (**b**, **d**). All images (**a**–**d**) were obtained using field microscopy with µM/pixel calibration. ×400 magnification (**a**–**c**); ×100 magnification (**d**). Representative analysis for ACPs evaluated in the study (*n* = 7). ACP, adamantinomatous craniopharyngioma

The Bioverso Test chemoresistance platform utilizes a mini-tumor or micro spheroid model three-dimensional cell aggregates derived from tumor tissue for in vitro drug screening. In our studies, micro spheroid cultures treated with temozolomide D2 (500 µM) demonstrated increased ACP tumor cell “spreading,” as shown by volumetric analysis indicating reduced micro spheroid area (Fig. 3b, d) when compared with untreated controls (Fig. 3a, c). These findings reflect the following: (i) effective intratumoral penetration of the chemotherapeutic agent, (ii) dispersion or loss of cell cohesion, (iii) reduced adhesion of clonogenic tumor cells, and (iv) temozolomide-induced cytotoxicity.

**Fig. 4:**
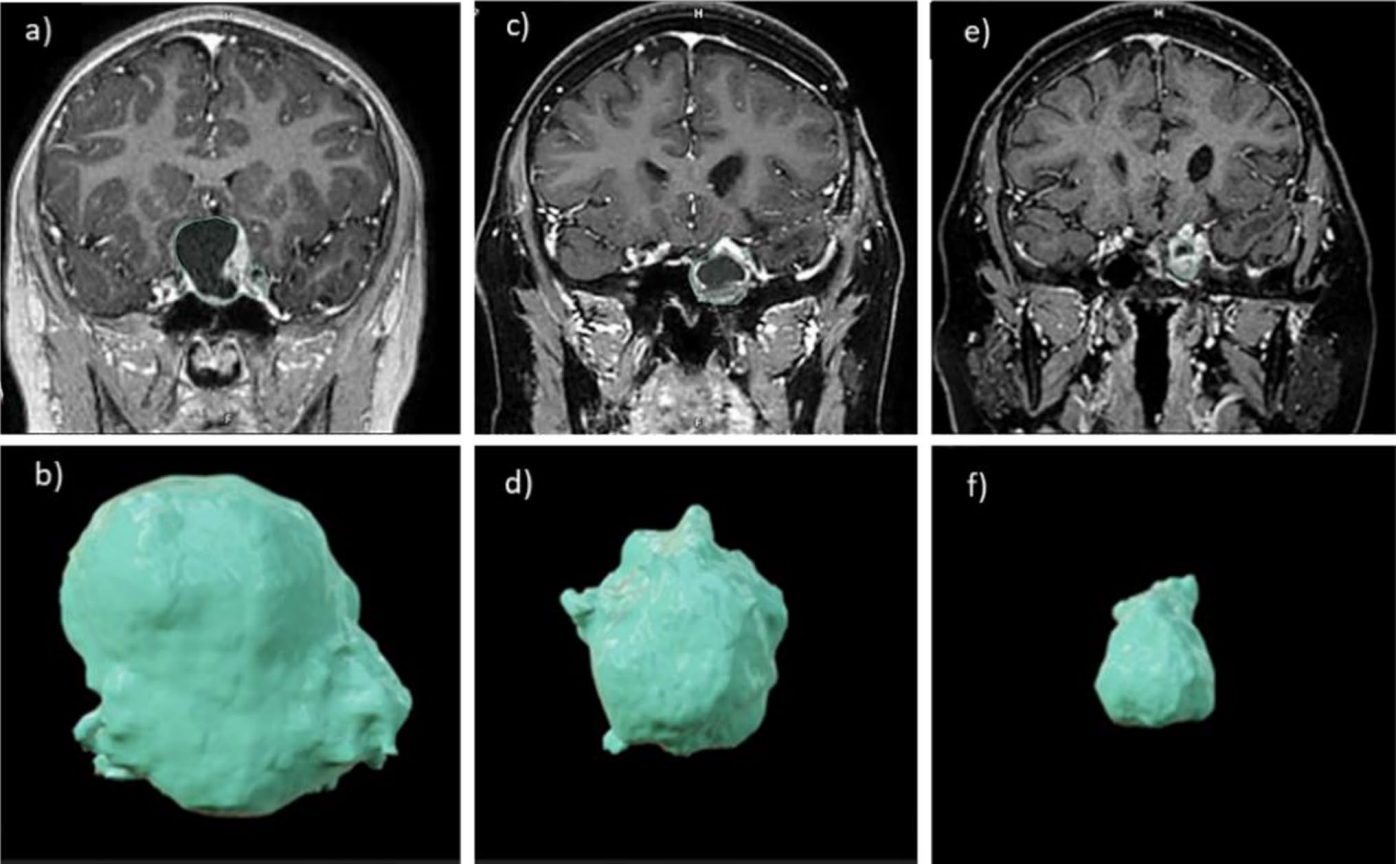
Brain MRI at diagnosis of ACP; **b** lesion volume at diagnosis, approximately 15 cm^3^; **c** MRI at the beginning of chemotherapy; **d** lesion volume of approximately 7 cm^3^ at chemotherapy initiation; **e** MRI following 12 cycles of temozolomide; and **f** lesion volume of approximately 3.5 cm^3^ after 12 cycles. ACP, adamantinomatous craniopharyngioma

In all ACP cases (*n* = 7/50), micro spheroids exhibited high sensitivity to temozolomide at 500 µM (Fig. 3). Based on these findings, a patient with recurrent ACP who had previously undergone two neurosurgical procedures and radiotherapy was selected for temozolomide therapy (Fig. 4).

### Illustrative case

In 2016, a 7-year-old boy presented with divergent strabismus, progressive visual impairment, reduced left visual acuity, and headache. Brain MRI showed a cystic, partially calcified suprasellar mass measuring 15 cm^3^ with enhancement (Fig. 4), suggestive of craniopharyngioma. Ophthalmologic evaluation revealed right-sided amaurosis and a 40% reduction in left visual acuity. Endocrinological evaluation confirmed panhypopituitarism.

### Postoperative course

The patient underwent partial resection via a transsphenoidal approach. A sphenoid mucosal flap was placed at closure. The postoperative course was good; however, a cerebrospinal fluid fistula developed and was resolved by repositioning the flap. Histopathology confirmed ACP.

## Radiological follow-up and clinical evolution

The patient underwent 3D volumetric modulated arc therapy radiotherapy 2 months after surgery, totaling 54 Gy across 30 fractions of 1.8 Gy each. Following radiotherapy, imaging revealed a small residual lesion at the primary site, which remained stable until 2020. After discontinuing follow-up, the patient presented to a local hospital with worsening left visual acuity and recurrent headaches. MRI revealed progression of the predominantly cystic residual lesion. In November 2023, the patient underwent a second partial resection. Postoperatively, he was referred back to our hospital. MRI showed a solid-cystic mass involving the left cavernous sinus, compressing the hypothalamus and extending into the sphenoid sinus, with an estimated volume of 7 cm^3^.

### Chemotherapy

Beginning in April 2024, the patient received 12 cycles of temozolomide (200 mg/m^2^), administered orally for 5 consecutive days every 28 days. Treatment was well tolerated, with no observed toxicity or delays in administration. Mild nausea was effectively managed with antiemetics. Visual assessments, including Snellen tests and visual evoked potential (VEP) evaluations, were performed before the first cycle and after the tenth cycle to monitor changes in visual function.

### Post-treatment course

Three months after chemotherapy, imaging showed an approximately 50% reduction in total lesion volume (from 7 to 3.5 cm^3^) (Fig. 4). The patient reported subjective improvement in visual acuity, although this was not corroborated by Snellen or VEP testing. The patient retains light perception in the left eye and none in the right.

## Discussion

ACP is the most prevalent neoplasm of the hypothalamic–pituitary axis in childhood [21, 43]. Despite its WHO grade I classification, ACP frequently leads to profound neurological and endocrine sequelae due to involvement of critical structures, including the hypothalamus, circle of Willis, optic pathways, and pituitary stalk.

Since Dean Lewis’s first successful surgical resection in 1909 [42], numerous surgical techniques have evolved to achieve gross total resection while minimizing morbidity and preserving vital anatomy. Advances in surgical methodology have enabled more extensive resections while protecting the diencephalic–pituitary structures. Transsphenoidal approaches facilitate substantial tumor removal while preserving the hypophyseal arteries, reducing endocrine deficits. The use of pedicled nasal mucosal flaps has significantly reduced cerebrospinal fluid leakage, a previously common complication [9, 31]. However, ACP’s rarity limits the number of medical centers capable of safely resecting large lesions without increasing morbidity and mortality. The extensive calcifications characteristic of ACPs complicate surgical resection and pose a significant risk of hypothalamic injury. Saint-Rose et al. [44] describe strategies ranging from attempts at total resection to more conservative approaches, such as partial resection, particularly when the hypothalamus is involved, followed by adjuvant radiotherapy. Nevertheless, radiotherapy, while preserving structural integrity, can compromise hormonal function and does not guarantee prevention of recurrence. Complete surgical excision remains the standard, although it carries long-term risks that can substantially affect quality of life [24, 55].

The optimal therapeutic strategy for ACP remains highly debated. Even after neurosurgical intervention, recurrence rates remain high, significantly affecting both morbidity and mortality [39, 53]. Combining surgery, radiotherapy, and intratumoral drug administration aims to maximize tumor control while minimizing complications; however, recurrence remains frequent. Beuriat et al. [5] reported a 26% recurrence rate in 26 ACP cases, and Choux et al. [11] found a 29.7% recurrence rate in 474 cases. Even gross total resection demonstrated a 19% recurrence rate, compared with 56.6% in cases with residual tumor, indicating limited progress despite surgical advances.

To reduce morbidity while controlling tumor growth, alternative pharmacologic strategies have emerged. Bleomycin, proposed by Takahashi et al. [45], demonstrated rapid resolution of cystic components and partial reduction of calcified areas [8]. However, bleomycin extravasation has been associated with significant complications. Interferon-alpha subsequently emerged as a therapeutic alternative, first administered subcutaneously by Jakacki et al. [29] and later via intralesional injection by Cavalheiro et al. [7], exhibiting antineoplastic activity without the systemic toxicity observed with bleomycin. Ierardi et al. [28] further validated its pro-apoptotic and antiproliferative properties in ACP models. Pettorini et al. [41] identified elevated concentrations of alpha-defensins 1 and 3 in cystic fluid, suggesting novel molecular targets. Alpha-defensins play key roles in innate immunity and inflammatory processes and have been shown to attenuate inflammation following intralesional interferon-alpha therapy. Genomic, proteomic, and transcriptomic studies have identified elevated inflammatory cytokines, including IL-6, in ACP pathogenesis, highlighting potential targeted therapies such as tocilizumab or siltuximab [18, 40].

Coy et al. [13] and Witt et al. [52] demonstrated that ACPs express programmed death protein-1 (PD-1) and its ligand PD-L1. Therapeutic inhibition of the PD-1/PD-L1 interaction with immune checkpoint inhibitors such as nivolumab or pembrolizumab may therefore represent a viable targeted treatment strategy.

The chemoresistance platform used in this study (*Bioverso Test*) employed a microspheroid model of three-dimensional cell aggregates for in vitro drug screening. This approach offers superior representation of intratumoral heterogeneity compared with conventional two-dimensional cultures and more accurately recapitulates in vivo tumor physiology, including cell–cell adhesion, hypoxia, morphology, and drug-penetration gradients [30, 48, 56]. Using this platform, seven freshly resected ACP specimens, confirmed by a neuropathologist, were analyzed after microspheroid formation. All ACP specimens demonstrated high sensitivity to temozolomide, indicating its cytotoxicity and ability to penetrate microspheroids, induce dispersion, disrupt cellular cohesion, and reduce adhesion among clonogenic ACP cell clusters.

Temozolomide is an alkylating chemotherapeutic agent widely used in high-grade glioma treatment because it can cross the blood–brain barrier. It acts directly on DNA, undergoing hydrolysis in the stomach and bloodstream to form the active metabolite 5-(3-methyltriazen-1-yl)-imidazole-4-carboxamide (MTIC). MTIC transfers methyl groups to DNA, leading to cell cycle arrest and tumor cell death. The DNA repair enzyme O6-methylguanine-DNA methyltransferase (MGMT) can reverse this damage; therefore, tumors with high MGMT expression exhibit reduced sensitivity to temozolomide. In our seven ACPs, tumor cells demonstrated minimal MGMT expression (≤ 10% immunoreactivity). Consistently, Zuhur et al. [57] retrospectively analyzed 23 ACP samples from patients aged 5–61 years and reported absent MGMT activity in 22 cases and weak activity in one. DNA repair pathways are fundamental to the survival and genomic stability of all living cells, including those comprising benign neoplasms. O⁶-Methylguanine-DNA methyltransferase (MGMT) is constitutively expressed in most normal tissues, where it functions to prevent the accumulation of mutagenic O⁶-methylguanine adducts generated by endogenous metabolic processes and environmental alkylating agents. In benign tumors, preserved MGMT expression—typically associated with an unmethylated promoter—represents the baseline physiological state, reflecting intact cellular defense mechanisms rather than an absence of selective pressure for DNA repair [12].

MGMT expression has been specifically investigated in craniopharyngiomas because of its potential therapeutic implications. Immunohistochemical analyses indicate that adamantinomatous craniopharyngiomas frequently exhibit low or absent MGMT protein expression, whereas data regarding MGMT promoter methylation remain limited and inconsistent across studies. [57]

Observations from pituitary adenomas—another common category of benign CNS tumors—further illustrate the heterogeneity of MGMT regulation in non-malignant neoplasms. Although the majority of pituitary adenomas demonstrate preserved MGMT expression, a subset of clinically aggressive or treatment-refractory tumors shows reduced MGMT expression or promoter methylation. In these selected cases, low MGMT expression has been validated as a key predictive biomarker of response to temozolomide (TMZ) [37]. These findings underscore that MGMT is not only expressed in benign tumors but also represents the principal determinant of TMZ efficacy when systemic therapy is indicated.

In the clinical case presented in this study, the patient treated with temozolomide had previously undergone two tumor resections and radiotherapy but experienced recurrence. Clinically, the patient exhibited panhypopituitarism and bilateral amaurosis, with tumor infiltration into the left cavernous sinus and sphenoid sinus. Treatment with 12 cycles of temozolomide resulted in a significant reduction of both cystic and solid components by more than 50%. A retrospective analysis of tumor tissue demonstrated negative MGMT expression. However, a fresh tumor was not available for chemoresistance testing in this patient. Total tumor volume decreased from 7 cm^3^ at chemotherapy initiation to 3.5 cm^3^ at completion.

During the early 2000 s, several in vitro chemotherapy response assays were developed to predict tumor sensitivity or resistance to chemotherapeutic agents [36]. Advances in personalized medicine have facilitated the integration of precision oncology approaches into the management of selected tumor types, enabling more tailored and potentially effective therapeutic strategies [15, 36, 54].

Chemoresistance assays identify agents to which a tumor is resistant, whereas chemosensitivity assays determine drugs capable of eliciting a therapeutic response [14]. These assays evaluate tumor growth inhibition in response to single agents or drug combinations. Chemoresistance testing specifically emphasizes negative predictive value, reflecting the assay’s ability to identify ineffective agents. Weisenthal and Kern [51] reported that the negative predictive value of in vitro drug response assays ranges from 90 to 99%, whereas the positive predictive value is lower (between 50 and 70%). Consequently, drug resistance prediction is more reliable for identifying agents likely to be ineffective than for determining those that will be clinically efficacious.

Drug response assessment systems leverage the genetic and phenotypic characteristics of individual tumors. By identifying interindividual differences in tumor drug response, personalized therapeutic regimens can be developed, optimizing efficacy while reducing unnecessary treatment costs [22].

Several studies have demonstrated promising outcomes using drug response assessment tests. Mehta et al. [38] evaluated breast tumors for conventional resistance to anthracycline and cyclophosphamide or to cyclophosphamide, methotrexate, and 5-fluorouracil. Patients exhibiting extreme or intermediate in vitro resistance (*n* = 55) experienced faster disease progression (48 months) compared with those demonstrating low in vitro resistance (*n* = 41, 100 months; *p* = 0.022), with a significant impact on overall survival (*p* = 0.011). In advanced ovarian tumors, Holloway et al. [25] reported that in vitro resistance correlated with increased disease progression and mortality when patients were treated with standard platinum-based regimens. Similarly, Loizzi et al. [35] observed that patients with recurrent ovarian cancer (*n* = 50) receiving therapy guided by chemoresistance testing achieved an overall response rate of 65% compared with 35% in patients treated empirically. To date, no studies have evaluated chemoresistance testing for CNS tumors in pediatric populations.

Personalized chemoresistance testing enhances the probability of therapeutic success by minimizing exposure to ineffective agents, reducing adverse drug reactions, and increasing the likelihood of tumor control or remission. Economically, this approach reduces costs associated with unnecessary medications, prolonged hospitalizations due to treatment-related complications, and palliative care resulting from therapeutic failure [47]. Environmentally, limiting the use of ineffective agents reduces the production and disposal of pharmaceuticals, thereby mitigating chemical and pharmaceutical waste [16].

In this context, the present study employed an in vitro chemoresistance platform that demonstrated significant TMZ sensitivity in ACP specimens, consistent with the clinical response observed in the illustrative case.

These findings raise important questions regarding new therapeutic strategies for pediatric ACP. Specifically, could the placement of an Ommaya reservoir for cyst drainage combined with intralesional temozolomide administration serve as an alternative for large cystic lesions, similar to established bleomycin and interferon-alpha protocols?

An additional question arises regarding the management of patients undergoing partial resection of ACPs: could temozolomide be administered before or concomitantly with radiotherapy? For children under 3 years of age who undergo partial resection, chemotherapy could serve as a bridge while awaiting radiotherapy. In cases diagnosed primarily based on growth deficits without other neurological symptoms, trial chemotherapy may offer a means of delaying or avoiding surgical intervention. Further studies and prospective clinical trials are needed to clarify the potential role of temozolomide in these scenarios.

## Conclusion

Our findings suggest that temozolomide represents a promising therapeutic option for ACP, particularly in cases of post-radiation recurrence and tumors exhibiting low MGMT expression. The integration of personalized chemoresistance testing may facilitate a shift from empiric multimodal treatment toward precision-based oncology. Multicenter clinical trials are essential to validate these preliminary observations and to establish temozolomide as part of the therapeutic strategy for pediatric craniopharyngiomas.

## Data Availability

This research did not generate additional datasets.

## References

[1] Adamson TE, Wiestler OD, Kleihues P, Yasargil MG (1990) Correlation of clinical and pathological features in surgically treated craniopharyngiomas. J Neurosurg 73:12–17. 10.3171/jns.1990.73.1.0012 2352012 10.3171/jns.1990.73.1.0012

[2] Albright AL, Hadjipanayis CG, Lunsford LD, Kondziolka D, Pollack IF, Adelson PD (2005) Individualized treatment of pediatric craniopharyngiomas. Childs Nerv Syst 21:649–654. 10.1007/s00381-005-1185-6 15931512 10.1007/s00381-005-1185-6

[3] An W, Li S, An Y, Lin Z (2025) Molecular subtypes of adamantinomatous craniopharyngiomas. Neuro Oncol 27:1180–1192. 10.1093/neuonc/noaf030 39898434 10.1093/neuonc/noaf030 PMC12187517

[4] Apps JR, Martinez-Barbera JP (2016) Molecular pathology of adamantinomatous craniopharyngioma: review and opportunities for practice. Neurosurg Focus 41:E4. 10.3171/2016.8.FOCUS16307 27903120 10.3171/2016.8.FOCUS16307

[5] Beuriat P-A, Szathmari A, Di Rocco F, Villanueva C, Bazus SC, Veyrie M, Mottolese C (2025) Craniopharyngiomas in children: considerations from a recent series of 26 patients treated in Lyon. Childs Nerv Syst 41:169 40285890 10.1007/s00381-025-06815-3 PMC12033113

[6] Bianchi F, Benato A, Massimi L (2022) Treatment of cystic craniopharyngiomas: an update. Adv Tech Stand Neurosurg 45:139–176. 10.1007/978-3-030-99166-1_4 35976449 10.1007/978-3-030-99166-1_4

[7] Cavalheiro S, Dastoli PA, Silva NS, Toledo S, Lederman H, da Silva MC (2005) Use of interferon-alpha in intratumoral chemotherapy for cystic craniopharyngioma. Childs Nerv Syst 21:719–724. 10.1007/s00381-005-1226-1 16133276 10.1007/s00381-005-1226-1

[8] Cavalheiro S, Sparapani FV, Franco JO, da Silva MC, Braga FM (1996) Use of bleomycin in intratumoral chemotherapy for cystic craniopharyngioma. J Neurosurg 84:124–126. 10.3171/jns.1996.84.1.0124 8613819 10.3171/jns.1996.84.1.0124

[9] Cavallo LM, Somma T, Solari D, Iannuzzo G, Frio F, Baiano C, Cappabianca P (2019) Endoscopic endonasal transsphenoidal surgery: history and evolution. World Neurosurg 127:686–694. 10.1016/j.wneu.2019.03.048 31266131 10.1016/j.wneu.2019.03.048

[10] Choux M, Lena G, Genitori L (1991) Le craniopharyngiome de l’enfant. Neurochirurgie 37:7–10

[11] Choux M, Lena G, Genitori L (1991) Le craniopharyngiome de l’enfant. Les récidives. Neurochirurgie 37:132–145

[12] Christmann M, Verbeek B, Roos WP, Kaina B (2011) O(6)-methylguanine-DNA methyltransferase (MGMT) in normal tissues and tumors: enzyme activity, promoter methylation and immunohistochemistry. Biochim Biophys Acta 1816(2):179–190. 10.1016/j.bbcan.2011.06.002 21745538 10.1016/j.bbcan.2011.06.002

[13] Coy S, Rashid R, Lin JR, Du Z, Donson AM, Hankinson TC, Foreman NK, Manley PE, Kieran MW, Reardon DA, Sorger PK, Santagata S (2018) Multiplexed immunofluorescence reveals potential PD-1/PD-L1 pathway vulnerabilities in craniopharyngioma. Neuro Oncol 20:1101–1112. 10.1093/neuonc/noy035 29509940 10.1093/neuonc/noy035 PMC6280314

[14] Cree IA (2009) Chemosensitivity and chemoresistance testing in ovarian cancer. Curr Opin Obstet Gynecol 21:39–43. 10.1097/GCO.0b013e32832210ff 19125002 10.1097/GCO.0b013e32832210ff

[15] Economopoulou P, Dimitriadis G, Psyrri A (2015) Beyond BRCA: new hereditary breast cancer susceptibility genes. Cancer Treat Rev 41:1–8. 10.1016/j.ctrv.2014.10.008 25467110 10.1016/j.ctrv.2014.10.008

[16] Freitas LAA, Radis-Baptista G (2021) Pharmaceutical pollution and disposal of expired, unused and unwanted medicines in Brazil. J Xenobiot 11:61–76. 10.3390/jox11020005 34069823 10.3390/jox11020005 PMC8162542

[17] Gritsch D, Santagata S, Brastianos PK (2024) Integrating systemic therapies into the multimodality therapy of patients with craniopharyngioma. Curr Treat Options Oncol 25:261–273. 10.1007/s11864-023-01156-2 38300480 10.1007/s11864-023-01156-2 PMC11203386

[18] Grob S, Mirsky DM, Donson AM, Dahl N, Foreman NK, Hoffman LM, Hankinson TC, Mulcahy Levy JM (2019) Targeting IL-6 as a potential treatment for primary cystic craniopharyngioma. Front Oncol 9:791. 10.3389/fonc.2019.00791 31497533 10.3389/fonc.2019.00791 PMC6712354

[19] Gupta DK, Ojha BK, Sarkar C, Mahapatra AK, Mehta VS (2006) Recurrence in craniopharyngiomas: analysis of clinical and histological features. J Clin Neurosci 13:438–442. 10.1016/j.jocn.2005.05.013 16678722 10.1016/j.jocn.2005.05.013

[20] Gupta S, Bi WL, Larsen AG, Abdulmohsen S, Abedalthagafi M, Dunn IF (2018) Craniopharyngioma: a roadmap for scientific translation. Neurosurg Focus 44:E12. 10.3171/2018.3.FOCUS1861 29852761 10.3171/2018.3.FOCUS1861

[21] Hankinson TC, Fields EC, Torok MR, Beaty BL, Handler MH, Foreman NK, O’Neill BR, Liu AK (2012) Limited utility despite accuracy of the national SEER dataset for the study of craniopharyngioma. J Neurooncol 110:271–278. 10.1007/s11060-012-0966-5 22915191 10.1007/s11060-012-0966-5

[22] Haroun RI, Clatterbuck RE, Gibbons MC, Burger PC, Parker R, Fruehauf JP, Brem H (2002) Extreme drug resistance in primary brain tumors: in vitro analysis of 64 specimens. J Neurooncol 58:115–123. 10.1023/A:1016049111941 12164682 10.1023/a:1016049111941

[23] Haupt R, Magnani C, Pavanello M, Caruso S, Dama E, Garre ML (2006) Epidemiological aspects of craniopharyngioma. J Pediatr Endocrinol Metab 19(Suppl 1):289–293 16700303

[24] Hoffman HJ, Da Silva M, Humphreys RP, Drake JM, Smith ML, Blaser SI (1992) Aggressive surgical management of craniopharyngiomas in children. J Neurosurg 76:47–52. 10.3171/jns.1992.76.1.0047 1727168 10.3171/jns.1992.76.1.0047

[25] Holloway RW, Mehta RS, Finkler NJ, Li KT, McLaren CE, Parker RJ, Fruehauf JP (2002) Association between in vitro platinum resistance in the EDR assay and clinical outcomes in ovarian cancer. Gynecol Oncol 87:8–16. 10.1006/gyno.2002.6797 12468336 10.1006/gyno.2002.6797

[26] Hölsken A, Gebhardt M, Buchfelder M, Fahlbusch R, Blümcke I, Buslei R (2011) EGFR signaling regulates tumor cell migration in craniopharyngiomas. Clin Cancer Res 17:4367–4377. 10.1158/1078-0432.CCR-10-2811 21562037 10.1158/1078-0432.CCR-10-2811

[27] Hölsken A, Sill M, Merkle J, Schweizer L, Buchfelder M, Flitsch J, Fahlbusch R, Metzler M, Kool M, Pfister SM, von Deimling A, Capper D, Jones DT, Buslei R (2016) Adamantinomatous and papillary craniopharyngiomas are characterized by distinct epigenomic as well as mutational and transcriptomic profiles. Acta Neuropathol Commun 4:20. 10.1186/s40478-016-0287-6 26927026 10.1186/s40478-016-0287-6 PMC4770705

[28] Ierardi DF, Fernandes MJS, Silva IR, Thomazini-Gouveia J, Silva NS, Dastoli PA et al (2007) Apoptosis in alpha-interferon intratumoral chemotherapy for cystic craniopharyngiomas. Childs Nerv Syst 23:1041–1046. 10.1007/s00381-007-0409-3 17593372 10.1007/s00381-007-0409-3

[29] Jakacki RI, Cohen BH, Jamison C, Mathews VP, Arenson E, Longee DC, Hilden J, Cornelius A, Needle M, Heilman D, Boaz JC, Luerssen TG (2000) Phase II evaluation of interferon-alpha-2a for progressive or recurrent craniopharyngiomas. J Neurosurg 92:255–260. 10.3171/jns.2000.92.2.0255 10659012 10.3171/jns.2000.92.2.0255

[30] A, Vainer B, Ibsen P, Harting H, et al (2017) Short-term spheroid culture of primary colorectal cancer cells for personalized cancer medicine. PLoS One 12:e0183074. 10.1371/journal.pone.0183074 10.1371/journal.pone.0183074 PMC5587104 28877221

[31] Kassam AB, Gardner PA, Snyderman CH, Carrau RL, Mintz AH, Prevedello DM (2008) Expanded endonasal approach for resection of midline suprasellar craniopharyngiomas: a classification based on the infundibulum. J Neurosurg 108:715–728. 10.3171/JNS/2008/108/4/0715 18377251 10.3171/JNS/2008/108/4/0715

[32] Kickingereder P, Maarouf M, El Majdoub F, Fuetsch M, Lehrke R, Wirths J, Luyken K, Schomaecker K, Treuer H, Voges J, Sturm V (2012) Intracavitary brachytherapy using stereotactically applied phosphorus-32 colloid for treatment of cystic craniopharyngiomas in 53 patients. J Neurooncol 109:365–374. 10.1007/s11060-012-0902-8 22717668 10.1007/s11060-012-0902-8

[33] Klimo P, Venable GT, Boop FA, Merchant TE (2015) Recurrent craniopharyngioma after conformal radiation in children and the burden of treatment. J Neurosurg Pediatr 15:499–505. 10.3171/2014.10.PEDS14384 25700121 10.3171/2014.10.PEDS14384

[34] Larkin SJ, Ansorge O (2013) Pathology and pathogenesis of craniopharyngiomas. Pituitary 16:9–17. 10.1007/s11102-012-0418-4 22886701 10.1007/s11102-012-0418-4

[35] Loizzi V, Chan JK, Osann K, Cappuccini F, DiSaia PJ, Berman ML (2003) Survival outcomes in recurrent ovarian cancer treated with chemoresistance assay-guided therapy. Am J Obstet Gynecol 189:1301–1307. 10.1067/s0002-9378(03)00629-x 14634558 10.1067/s0002-9378(03)00629-x

[36] Marcolin JC, Lichtenfels M, da Silva CA, de Farias CB (2023) Gynecologic and breast cancers: updates in chemoresistance and chemosensitivity tests. Curr Probl Cancer 47:100996. 10.1016/j.currproblcancer.2023.100996 37467541 10.1016/j.currproblcancer.2023.100996

[37] McCormack AI, McDonald KL, Gill AJ et al (2009) Low O6-methylguanine-DNA methyltransferase (MGMT) expression and response to temozolomide in aggressive pituitary tumours. Clin Endocrinol (Oxf) 71(2):226–233. 10.1111/j.1365-2265.2008.03487.x 19067722 10.1111/j.1365-2265.2008.03487.x

[38] Mehta RS, Bornstein R, Yu IR, Parker RJ, McClaren CE, Nguyen KP, Fruehauf JP (2001) Breast cancer survival and in vitro tumor response in the extreme drug resistance assay. Breast Cancer Res Treat 66:225–237. 10.1023/A:1010604502627 11510694 10.1023/a:1010604502627

[39] Muller HL (2008) Childhood craniopharyngioma: recent advances in diagnosis, treatment and follow-up. Horm Res 69:193–202. 10.1159/000113019 18204266 10.1159/000113019

[40] Nellan A, Lester McCully CM, Garcia RC, Jayaprakash N, Widemann BC, Lee DW, Warren KE (2018) Improved CNS exposure to tocilizumab after cerebrospinal fluid versus intravenous administration in rhesus macaques. Blood 132:662–666. 10.1182/blood-2018-05-846428 29954750 10.1182/blood-2018-05-846428 PMC6086204

[41] Pettorini BL, Inzitari R, Massimi L, Tamburrini G, Caldarelli M, Fanali C, Cabras T, Messana I, Castagnola M, Di Rocco C (2010) The role of inflammation in the genesis of the cystic component of craniopharyngiomas. Childs Nerv Syst 26:1779–1784. 10.1007/s00381-010-1245-4 20668862 10.1007/s00381-010-1245-4

[42] Raimondi AJ (1991) Le craniopharyngiome de l’enfant – Introduction. Neurochirurgie 37:11

[43] Russel DS, Rubinstein LJ (1989) Pathology of tumors of the nervous system, 5th ed. Williams & Wilkins, Baltimore, pp 695–702

[44] Sainte-Rose C, Puget S, Wray A, Zerah M, Grill J, Brauner R, Boddaert N, Pierre-Kahn A (2005) Craniopharyngioma: the pendulum of surgical management. Childs Nerv Syst 21:691–695. 10.1007/s00381-005-1209-2 16078079 10.1007/s00381-005-1209-2

[45] Takahashi H, Nakazawa S, Shimura T (1985) Evaluation of postoperative intratumoral injection of bleomycin for craniopharyngioma in children. J Neurosurg 62:120–127. 10.3171/jns.1985.62.1.0120 2578064 10.3171/jns.1985.62.1.0120

[46] Tritos NA (2015) Is there a role for targeted medical therapies in patients with craniopharyngiomas? Future Oncol 11:3221–3223. 10.2217/fon.15.233 26562628 10.2217/fon.15.233

[47] Tsimberidou AM, Fountzilas E, Nikanjam M, Kurzrock R (2020) Precision cancer medicine: evolution of the treatment paradigm. Cancer Treat Rev 86:102019. 10.1016/j.ctrv.2020.102019 32251926 10.1016/j.ctrv.2020.102019 PMC7272286

[48] Vinci M, Gowan S, Boxall F, Patterson L, Zimmermann M, Court W et al (2012) Advances in three-dimensional tumor spheroid-based assays for drug evaluation. BMC Biol 10:29. 10.1186/1741-7007-10-29 22439642 10.1186/1741-7007-10-29 PMC3349530

[49] Wang K-C, Hong SH, Kim S-K, Cho BK (2005) Origin of craniopharyngiomas: implications on the growth pattern. Childs Nerv Syst 21:628–634. 10.1007/s00381-005-1203-8 16059733 10.1007/s00381-005-1203-8

[50] Wang X, Zhao C, Lin J, Liu H, Zeng Q, Chen H et al (2024) Multi-omics analysis of adamantinomatous craniopharyngiomas reveals distinct molecular subgroups with prognostic and treatment response significance. Chin Med J (Engl) 137:859–870. 10.1097/CM9.0000000000002774 37565822 10.1097/CM9.0000000000002774 PMC10997223

[51] Weisenthal LM, Kern DH (1991) Prediction of drug resistance in cancer chemotherapy: the Kern and DiSC assays. Oncology 5:93–103 1835882

[52] Witt DA, Donson AM, Amani V, Moreira DC, Sanford B, Hoffman LM, Handler MH, Levy JMM, Jones KL, Nellan A, Foreman NK, Griesinger AM (2018) Specific expression of PD-L1 in RELA-fusion supratentorial ependymoma. Pediatr Blood Cancer 65:e26960. 10.1002/pbc.26960 29350470 10.1002/pbc.26960 PMC5867234

[53] Yamada S, Fukuhara N, Oyama K, Takeshita A, Takeuchi Y, Ito J et al (2010) Surgical outcome in 90 patients with craniopharyngioma: an evaluation of transsphenoidal surgery. World Neurosurg 74:320–330. 10.1016/j.wneu.2010.06.014 21492566 10.1016/j.wneu.2010.06.014

[54] Yan J, Liu Z, Du S, Li J, Ma L, Li L (2020) Diagnosis and treatment of breast cancer in the precision medicine era. Methods Mol Biol 2204:53–61. 10.1007/978-1-0716-0904-0_5 32710314 10.1007/978-1-0716-0904-0_5

[55] Yasargil MG, Curcic M, Kis M, Siegenthaler G, Teddy PJ, Roth P (1990) Total removal of craniopharyngiomas. Approaches and long-term results in 144 patients. J Neurosurg 73:3–11. 10.3171/jns.1990.73.1.0003 2352020 10.3171/jns.1990.73.1.0003

[56] Zanoni M, Piccinini F, Arienti C, Zamagni A, Santi S, Polico R et al (2016) 3D tumor spheroid models for in vitro therapeutic screening: enhancing the biological relevance of data obtained. Sci Rep 6:19103. 10.1038/srep19103 26752500 10.1038/srep19103 PMC4707510

[57] Zuhur SS, Müslüman AM, Tanık C, Karaman O, Oztürk FY, Ozderya A, Ozkayalar H, Aydın Y, Altuntaş Y (2011) MGMT immunoexpression in adamantinomatous craniopharyngiomas. Pituitary 14:323–327. 10.1007/s11102-011-0297-0 21318329 10.1007/s11102-011-0297-0

